# 

**Published:** 1888-11

**Authors:** 


					﻿Volume LVII.J
NOVEMBER, 1888.
I Number 5.

T	..
foisOπfeLi üS><
ini mi j ii yy£ w
1 dKfil fil 11CI N
EDiToa:
S. J. JONES, M.D., LL.D.
THE CHICAGO MEDICAL PRESS ASSOCIATION.
RAND, McNALLY & CO., PRINTERS.	I
Entered at the Poat Office at Chicago, for tranβmiaaion through the mail* aa aecond-olaaa matter.
				

